# Effectiveness of Physical Activity Interventions on Return to Work After a Cancer Diagnosis: A Systematic Review and Meta-analysis

**DOI:** 10.1007/s10926-022-10052-9

**Published:** 2022-07-02

**Authors:** Têtê Norbert Wilson, Aboubakari Nambiema, Bertrand Porro, Alexis Descatha, Agnès Aublet-Cuvelier, Bradley Evanoff, Yves Roquelaure

**Affiliations:** 1 Univ Angers, CHU Angers, Univ Rennes, Inserm, EHESP, Irset (Institut de Recherche en Santé, environnement et travail) - UMR_S 1085, 49000 Angers, France; 2 Direction des Etudes et de Recherches, INRS (Institut National de Recherche Et de Sécurité), 1 rue du Morvan, CS60027, 54519 Vandœuvre-lès-Nancy, France; 3 Division of General Medical Sciences, Washington University School of Medicine, St. Louis, MO 63310 USA

**Keywords:** Intervention, Return to work, Cancer, Physical activity, Systematic review

## Abstract

**Abstract:**

*Purpose* The aim of this study was to assess the effectiveness of physical activity (PA) interventions on return to work (RTW) in cancer survivors, compared to usual care, and to determine the dose of PA needed to improve this outcome. *Methods* A systematic review and meta-analysis were conducted according to PRISMA guidelines. Six electronic databases including PubMed, Embase, Web of Science, CENTRAL, PsycINFO, and Scopus were searched to identify studies, and completed by a search of grey literature and health organization websites. Two authors performed screening, selection, and data extraction independently. Study and intervention characteristics were extracted and summarized. Pooled risk ratio (RR) was estimated using a weight random-effects model with 95% confidence intervals (CIs). *Results* A total of 2655 records were identified, of which 8 intervention studies were included. The sample size of the included studies varied between 41 and 240, giving a total of 1087 participants aged between 18 and 75 years. Compared with usual care, PA interventions had a significant positive effect on RTW among cancer survivors with a pooled RR of 1.29 (95% CI 1.17, 1.42). We found that PA interventions (aerobic and resistance exercises) with an exercise dose between 7.6 METs.h/week and 15 METs.h/week, consisting in 50–60 min per session of moderate to vigorous physical exercise, twice a week seems relevant in improving RTW. *Conclusions* Our results showed, with moderate quality evidence that PA interventions are more effective than usual care in increasing the rate of RTW in cancer survivors.

**Systematic Review Registration:**

PROSPERO Registration Number, CRD42020203614.

**Supplementary Information:**

The online version contains supplementary material available at 10.1007/s10926-022-10052-9.

## Background

Cancer is one of the leading causes of morbidity worldwide, with approximatively 19.3 million new cases diagnosed in 2020 [1]. Over the last few decades, advances in early detection and treatments have greatly contributed to the increased average survival of cancer patients.

Despite improved survival rates, it has been reported that cancer survivors usually experience long-term side effects from cancer and its treatment (e.g. cancer-related fatigue, pain, anxiety and depression) [2, 3]. These medical and psychological effects may become persistent, affecting the quality of life and work ability of cancer survivors (aged more than 18 years), and rendering it challenging to remain in or return to work (RTW) [4, 5]. Around 26 to 53% of cancer survivors will experience work loss and fail to RTW after diagnosis [6]. However, most of cancer survivors are motivated either to RTW or to be re-employed after treatment [7]. They regard returning to work as a symbol of full recovery and regaining a normal life [8]. Returning to work can also help maintain family income, improve self-esteem, sense of meaning, and health [8, 9].

Given the increasing number of cancer survivors at working age and the multiple challenges they face, there is a need to provide tailored programs supporting the RTW of cancer survivors [10]. Physical activity (PA) has been shown as an effective intervention to address some of the side effects from treatment [11], by decreasing fatigue and/or emotional distress levels and increasing the level of perceived quality of life of patients affected by cancer [12]. Based on these findings, several interventions including PA have been developed to help cancer survivors to RTW after cancer diagnosis [13–15]. Some systematic reviews of rehabilitation interventions revealed that PA could contribute to improving RTW rates [13–16], while another review showed that PA interventions were not more effective than care as usual [17]. These contradictory results could be explained by the fact that previous systematic reviews included several interventions namely psychosocial, vocational, educational and multidisciplinary interventions and did not investigate the specific effect of PA on RTW in cancer survivors [13–16]. Moreover, the conditions for implementing PA interventions in terms of content and delivery (e.g., period, setting and mode of delivery) remains little explored in these systematic reviews [13–15].

Finally, the exercise dose–response and the best type of exercise in terms of duration, frequency and intensity of PA required to improve RTW remain unclear, making it difficult to recommend a specific exercise protocol for cancer survivors in the RTW intervention programs [18].

To the best of our knowledge, no systematic review to date, has specifically evaluated the effectiveness and dose–response of PA interventions on RTW in cancer survivors. Therefore, we conducted this systematic review to assess the effectiveness of PA intervention on RTW in cancer survivors compared to usual care, and to determine the dose of PA needed to improve this outcome. Based on the results achieved, recommendations will be suggested for implementation of PA interventions to support RTW in cancer survivors.

## Methods

This review was conducted following the Preferred Reporting Items for Systematic review and Meta-Analysis (PRISMA) guidelines [19]. The review protocol was registered in the International Prospective Register of Systematic Reviews (PROSPERO) under number CRD42020203614.

### Eligibility Criteria

Studies were included in this systematic review if they met the following PICOS criteria (Population, Intervention, Comparator, Outcomes and Study design): (i) randomized controlled trials (RCTs) or non-randomized controlled trials (nRCTs), (ii) conducted on participants (working adults, aged ≥ 18 years old) diagnosed with cancer (all location) and who were in paid employment (employee or self-employed) at the time of diagnosis, (iii) including any type of PA as interventions, carried out in any setting (clinical setting, or at home), prior, during or after treatment, supervised or unsupervised, (iv) compared to usual care, and (v) assessed RTW as outcome (rates of RTW or time to RTW). The control group participants included patients receiving usual or standard care and who did not follow or participate in the PA intervention. We included studies without restriction on publication dates.

Studies were excluded if they: concerned retired cancer survivors or pediatric cancers (childhood and young adults’ cancers); did not have a control group; did not assess RTW as an outcome; and/ or were not meet the design of intervention studies (case reports, case series, editorial, reviews, cross-sectional, case control and cohort studies).

### Information Sources and Search Strategy

To identify records, the following electronic databases were consulted: PubMed, Embase, the Cochrane Library, Web of Science, PsycINFO, and Scopus. Unpublished and ongoing studies were identified by searching a clinical trial database (Clinical Trial Gov), a grey literature database (OpenGrey), health organization websites and internet search-engine databases, such as: European Agency for Safety and Health at Work (OSHA), American Society of Clinical Oncology (ASCO), the French National Cancer Institute (INCA) and Google Scholar. In Google Scholar, only the first 200 hits were selected after ordering the hits by relevance. In addition, the reference lists of included studies and previous systematic reviews were hand searched in order to identify additional relevant studies. Finally, two experts were contacted (by e-mail) based on their scientific expertise, and publications on the topic, to provide information of known published or unpublished studies that should be included in this review.

The search strategy was based on PubMed and adapted to the specificity of each database. Keywords related to cancer, PA and RTW were identified and selected from Mesh database and earlier systematic reviews [16, 17]. The relevant keywords in Medical Subject Heading “[Mesh]” and text word “[TW]” terms were connected with Boolean operators “AND”/” OR” to build the search query. Some search terms were truncated to include variations in word endings, spellings, and database indices. In Google Scholar, OSHA and ASCO databases, filters were applied to refine the search output. The search strategy was modified to fit the specifics of other databases. Two external librarians reviewed the research query to make it more relevant. All searches were conducted using English language terms.

Records were searched in all databases from inception to December 8, 2020 and updated on September 30, 2021. Detailed search strategies for each database are available in Supplementary Table A.

### Study Selection

Study selection was carried out using the Covidence systematic review software [20]. All study records identified in the search were downloaded and duplicates were removed. After removing the duplicates, studies were screened for inclusion/exclusion decisions in two stages. Two review authors (TNW and AN), independently screened titles and abstracts (*step 1*), and then full texts (*step 2*) of potentially relevant records. Discrepancies between authors were resolved through discussion to reach consensus.

### Data Extraction

A standard data extraction form was developed and trialed until data extractors reached convergence and agreement. Two review authors (TNW and AN) independently extracted data from included studies. The following data were extracted from each included study feature (author, year of publication, country, and study design); population characteristics (sample size, age, sex, and type of cancer), interventions characteristics (type of PA, frequency, duration, intensity, exercise dose, intervention length, area, period, and mode of delivery). Data regarding control group (e.g., standard care, or usual care), outcomes; and main findings were also extracted. All discordances between data extractors were resolved by discussion to reach consensus.

#### Exercise Dose Calculation

Exercise dose was estimated using metabolic equivalent for task (MET), where 1 MET equates to 3.5 mlO_2_/kg/min. The corresponding MET values for exercise intensity were coded according to the compendium of PA [21] if no more details related to their content were provided and study’s authors did not respond to requests for the missing data. Thus, 3.8 and 6 METs were respectively assigned to moderate and vigorous intensity of resistance exercise; strength-training exercise was coded 3.5 METs; warm-up and cool-down were estimated at 2.5 METs. Yoga and stretching activities were coded to 2.8 METs. For interventions comprising several types of exercises of different duration, the average duration of each exercise was computed to estimate exercise dose. The estimation of a targeted exercise dose was calculated as:

$$\mathrm{Weekly\; exercise\; dose }= {\sum }_{i=1}^{n}{\left(Intensity\right)}_{i}\times {\left(Duration\right)}_{i}\times {\left(Frequency\right)}_{i} \mathrm{in MET.h/week},$$

where one exercise session is composed of *i* PA, the intensity of PA *i* is in METs, the average duration of PA *i* is in hours, and frequency is the number of sessions per week.

#### Dealing with Missing Data

During data extraction, if there are missing data in studies, the study’s authors were contacted by e-mail using the contact details provided in the article to obtain data that were missing in their report, which we needed as input. Follow-up e-mails were sent two weeks later if responses were not received. If responses were still not received after the reminder, and to be exhaustive in our research, the studies were retained in the systematic review and used for the narrative synthesis.

### Risk of Bias Assessment in Individual Studies

The Navigation Guide risk of bias tool was used to assess the risk of bias across included studies [22]. It was developed according to the standard risk of bias assessment methods of the Cochrane Collaboration [23], the Agency for Healthcare Research and Quality (AHRQ) [24] and adapted specifically to systematic review in occupational health. The tool has been successfully applied in several systematic reviews [25–29] and used by the World Health Organization (WHO) and International Labor Organization (ILO) experts network [30, 31]. Nine domains of bias were included in the Navigation Guide for human studies. For each domain, the risk of bias rating was “low risk”; “probably low risk”; “probably high risk”; “high risk”; or “not applicable”. The risk of bias assessment was conducted on the individual study level and across the body of evidence for each study. Two review authors (TNW and AN) independently assessed the risk of bias for each study by outcome. When the authors’ individual assessment differed, disagreements were resolved by discussion to reach consensus.

### Outcomes Measures and Intervention Effect

The outcome considered in our study was RTW after cancer diagnosis (including prior, during or after treatment). The RTW included any return to full-time, or part-time employment, to previous or new employment, and to either the same or reduced role after a sick leave due to cancer [17]. It was measured as rate of RTW (binary outcome) or time to RTW (continuous outcome). The rate of RTW is defined as the proportion of patients who returned to work in each arm (intervention and control) at the endpoint, whilst the time to RTW is the number of days between reporting sick leave and any work resumption or the number of days on sick leave during the follow-up period. The risk ratios (RRs) were used as the measure of intervention effect (effect size). They were calculated from the reported values of outcomes. All estimates were reported with their 95% confidence interval (CI) or P value.

### Assessment of Heterogeneity

First, we decided whether or not studies were sufficiently homogeneous to be able to synthesize the results into meta-analysis (pooled effect size). Studies were sufficiently homogeneous when they had similar designs, similar intervention and comparator, and similar outcome measure. Statistical heterogeneity was also tested with I^2^ statistic [32]. Studies were statistically heterogenous if I^2^ was greater than 50%.

### Synthesis of Results (Data Synthesis)

First, a narrative synthesis of the results from included studies was performed following the Popay et al. [33] framework for narrative synthesis. Secondly, meta-analysis was conducted to estimate the overall effect of PA on RTW by pooling the RRs of each study according to the Mantel–Haenszel method. A random effects model was used to estimate the overall effect size.

#### Sensitivity Analyses

Two sensitivity analyzes were conducted excluding one arm of intervention for the 3-arms RCTs. A meta-regression was also performed from included studies that presented complete data on PA characteristics to assess the association between exercise dose and intervention effects (RRs). Statistical analyzes were performed using RevMan version 5.4 and R 4.1.1 software. Statistical significance was set at alpha 5% (P value < 0.05) for all results.

#### Publication Bias

According to Cochrane Collaboration, tests for funnel plot asymmetry should be used to judge concerns on publication bias when there are at least 10 studies included in the meta-analysis [34, 35]. If there are fewer than 10 studies, the power of the tests is too low to distinguish chance from real asymmetry [35]. In this case, the risk of publication bias was judged qualitatively.

### Quality of Evidence Assessment

The quality of evidence for the entire body of evidence was assessed using the Navigation Guide approach for grading the quality and strength across human studies [27]. The Navigation Guide is based on the Grading of Recommendations Assessment, Development and Evaluation (GRADE) approach [36] and adapted specifically to systematic review in occupational health. However, the Navigation Guide allows for rating evidence based on the following 8 domains: (i) risk of bias; (ii) inconsistency; (iii) indirectness of evidence; (iv) imprecision of the pooled estimate; and (v) possibility of publication bias; (vi) large magnitude of effect; (vii) dose–response; and (viii) residual confounding [37]. Two review authors (TNW and AN) independently assessed the quality of evidence for the entire body of evidence, and any disagreements were resolved by consensus. The entire body of evidence was graded using the three Navigation Guide standard quality of evidence ratings: “high”, “moderate” and “low” [25].

## Results

### Search Results and Study Selection

**Fig. 1:**
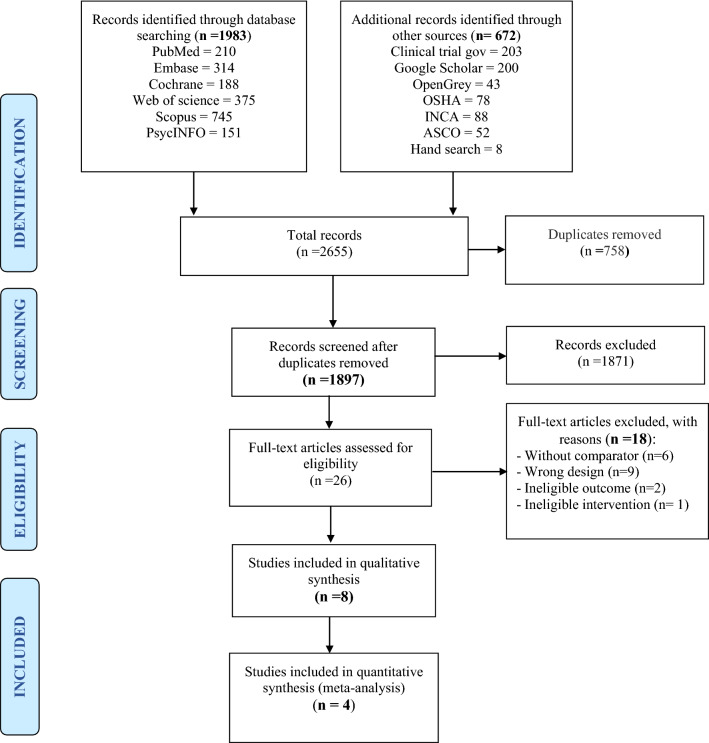
Flow diagram of study selection

Figure 1 presents the PRISMA flow diagram of the studies selection process. A total of 2655 records were identified including 1983 from database searches and 672 through other sources. Of these, 758 were duplicates and removed, leaving 1897 records for screening. The screening of titles and abstracts excluded 1871 studies that did not meet the eligibility criteria and included 26 articles for full-text stage. Following review of full-text articles, 18 studies were excluded and only 8 fulfilled all eligibility criteria for inclusion [38–45]. The reasons for exclusion of the 18 studies were: study with wrong design (n = 9), without control group (n = 6), without RTW outcomes (n = 2), and without PA intervention (n = 1).

### Description of Included Studies

In this section, the characteristics of studies, participants, and interventions, as well as comparators and outcomes are described and presented in Table 1.

**Table 1:** Characteristics of included studies in the systematic review

Study characteristics				Study population			
Study ID	Country of study	Study period (day/month/year)	Study design	Number of participants included	Age, range (years)	Sex (n)	Type of cancer (n)
Mijwel et al. [42]	Sweden	03/2013–07/2016	RCT	240	18–70	Women (240)	Breast (240)
Jong et al. [41]	Netherlands	NR	RCT	83	18–70	Women (83)	Breast stage I–III (83)
Ibrahim et al. [40]	Canada	2011–2015	RCT	59	18–45	Women (59)	Breast stage I–III (59)
Van Waart et al. [44]	Netherlands	03/2010–12/2012	RCT	230	51	Women (228) Men (2)	Breast (230)
Thijs et al. [43]	Netherlands	07/2001–06/2005	nRCT	110	18–65	Women (93) Men (17)	Breast (77) Colorectal (10) Hodgkin lymphoma (10) Others (13)
Burgio et al. [39]	USA	01/1996–01/2001	RCT	125	53–68	Men (125)	Prostate (125)
Rogers et al. [45]	USA	04/2006–04/2007	RCT	41	18–70	Women (41)	Breast stage I–IIIa (41)
Berglund et al. [38]	Sweden	NR	RCT	199	< 75	Women (199)	Breast (159) Ovarian (16) Others (24)

Characteristics of interventions										
Study ID	Type	Length^a^ (week)	Frequency (session/week)	Duration^b^ (min/session)	Intensity (MET)	Exercise dose^c^ (MET.h/week)	Area of delivery	Period of delivery	Delivery modes	Number of participants with outcome
Mijwel et al. [42]	RT-HIIT: resistance exercise followed by high-intensity interval training	16	2	60	3.80	7.60	Hospital	During and after treatment	Supervised	59
	AT-HIIT: aerobic exercise followed by high-Intensity interval training	16	2	60	6.00	12.00	Hospital	During and after treatment	Supervised	62
Jong et al. [41]	Dru yoga (breathing awareness, Energy block release, body awareness, relaxation)	12	1	75	2.80	3.55	Home and hospital	Before and during treatment	Supervised	40
Ibrahim et al. [40]	Exercise program: Cardiovascular exercise, strength training, endurance program and stretching program	12	3	NR	NR	NA	Home and hospital	After treatment	Supervised	29
Van Waart et al. [44]	Onco-Move: aerobic exercise	20	5	30	4.00	10.00	Home	During and after treatment	Not supervised	62
	OnTrack: resistance and aerobic exercise	20	2	50	9.00	15.00	Hospital	During and after treatment	Supervised	71
Thijs et al. [43]	Resistance and aerobic exercise	18	2	NR	6.00	NA	Hospital	After treatment	Supervised	72
Burgio et al. [39]	Strength exercise + Biofeedback assisted behavioral training	24	3	NR	NR	NA	Home and hospital	Before treatment	Not supervised	28
Rogers et al. [45]	Aerobic exercise + group discussions + counseling	12	3	50	3.80	9.50	Home and hospital	After treatment	Supervised	20
Berglund et al. [38]	Strength exercise + coping skills + information	7	2	NR	NR	NA	Hospital	After treatment	Supervised	87

Study ID	Comparator		Outcome assessment			
	Type	Number of participants with outcome	Definition and endpoint	Type	Effect measure	Studies’ conclusion (main results)
Mijwel et al. [42]	Usual care	52	RTW at 12 months (part time)	Secondary	Rate of RTW: 82.00% for RT-HIIT 91.00% for AT-HIIT 69.00% for usual care, P value = 0.020	At 12 months, both RT-HIIT and AT-HIIT displayed beneficial effect on RTW. The difference remained significant for AT-HIIT
Jong et al. [41]	Usual care	27	RTW at 6 months	Secondary	Rate of RTW: 53.00% for Yoga 23.00% for usual care	Possible favorable effects of the yoga program on early return to work in breast cancer survivors
Ibrahim et al. [40]	Usual care	30	RTW at 18 months	Secondary	Rate of RTW: 86.00%	The majority of participants returned to work
Van Waart et al. [44]	Usual care	64	RTW at 6 months (part time or full time)	Secondary	Rate of RTW: 83.00% for Onco-Track 79.00% for Onco-Move 61.00% for usual care, P value = 0.012	At 6 months, both intervention groups had significantly higher RTW rates than usual care (returned earlier to work than the control group). The Onco-track program was most effective in facilitating return to work
Thijs et al. [43]	Usual Care	38	RTW at 12 months and time to RTW (full time)	Primary	Rate of RTW: 78.00% for intervention group 66.00% for control group P value = 0.180	An oncologic rehabilitation program including high-intensity physical training results in substantial benefits effect by improving job resumption. No significance was found in RTW rate between groups
Burgio et al. [39]	Usual care	29	RTW at 6 months	Secondary	Rate of RTW: 78.60% for intervention group 79.30% for control group, P value: 0.950	The finding was not accompanied by differences in the proportion of men who returned to work or usual activities by the 6-month follow-up. No difference was found on RTW
Rogers et al. [45]	Usual care^d^	19	Time to RTW (number of sick days in past month)	Secondary	Log odds: For Intervention group = 0.20 For usual care = − 0.20, P value = 0.800 OR = 1.49	The intervention did not significantly change in number of sick days missed from work
Berglund et al. [38]	Usual care	89	RTW (work status) at 12 months	Primary	Rate of RTW: 74.60% for intervention group 60.90% for control group	No significant difference between intervention and control groups in the rate of RTW

*n* number of participants, *RCT* randomized controlled trials, *nRCT* non-randomized controlled trials, *USA* United States of America, *MET* Metabolic Equivalent of Task (Low: < 3MET, moderate: 3–6 MET and high: ≥ 6 MET), *RT-HIIT* Resistance Training combined with High-Intensity Interval Training, *AT-HIIT* Aerobic Training combined High-Intensity Interval Training, *RTW* return to work, *OR* odds ratio, *NA* not applicable, *NR* not reported

^a^Length of intervention, ^b^mean duration of exercise sessions, ^c^exercise dose = (Intensity) × (duration) × (frequency), ^d^Usual care: the usual care group was provided written materials related to physical activity obtained from the American Cancer. These materials were considered “usual care” because of their availability to the general public

#### Characteristics of Studies

Of the 8 included studies, 5 were published after 2010 [40–44], while the remaining 3 studies were published before (1994, 2006, and 2009) [38, 39, 45]. Seven studies were RCT [38–42, 44, 45], one was nRCT [43]. All included studies were conducted in high income countries, with most carried out in European countries (n = 5), particularly in the Netherlands [41, 41, 43, 44] and Sweden [38, 42]. The other 3 studies were conducted in North American countries: USA [38, 39] and Canada [40].

#### Characteristics of Participants

The sample size of included studies varied between 41 and 240 at inclusion, for a total of 1087 participants aged between 18 and 75 years. Most studies (n = 5) included women exclusively [38, 40–42, 45], one study included men exclusively [39], and the samples were mixed sex in 2 studies [43, 44]. More than half of the included studies (n = 5) involved breast cancer survivors exclusively [40–42, 44, 45]. Of these 5 studies, 3 specifically reported information of the tumor stage (from I to III). One study included prostate cancer survivors [39]. The remaining studies (n = 2) included participants with mixed cancer diagnoses: breast cancer (mainly), colorectal, upper gastrointestinal, Hodgkin lymphoma, ovarian, and other cancers [38, 43].

#### Intervention characteristics

Details on the interventions are presented in Table 1. Six studies included one intervention group [38–41, 43, 45], while 2 studies were 3-arm RCTs comparing 2 intervention groups to usual care [42, 44], leading to the inclusion of 10 intervention groups, each one compared with usual care.

The implementation of interventions varied widely. Of the 10 interventions, 5 were delivered in a hospital setting [38, 42–44], one was a home-based intervention [44], and 4 combined hospital and home-based sessions [39–41, 45]. The intervention length ranged from 7 to 24 weeks, and the majority (n = 8) were supervised. The mode of intervention delivery was reported as supervised when the PA was performed under the direct supervision of an instructor (by qualified or trained personnel including nurses, physical therapists, or physiotherapists) and non-supervised otherwise. Four periods were reported for the time that interventions were administered: (i) intervention delivered before treatment [39], (ii) intervention initiated 1–2 weeks before the therapy and finished during treatment [41], (iii) intervention began during treatment and finished a few weeks after treatment [42, 44], and (iv) intervention administered after the completion of treatment [38, 40, 43, 45]. The period before treatment includes the time from cancer diagnosis until the beginning of treatment.

The majority of PA interventions were reported with the FITT (Frequency, Intensity, Time, and Type of exercise) components of exercise. All studies reported the type of PA including resistance exercise, aerobic exercise, endurance exercise, strength training, and Yoga (see details in Supplementary Table B). Most of the interventions (n = 7) involved PA only [40–44]. Of these, 2 interventions included aerobic exercise [42, 44], one included resistance exercise [42], 2 included a combination of resistance and aerobic exercises [43, 44], and 2 included multicomponent exercises [40, 41]. In the remaining studies (n = 3), exercise was combined with other interventions [38, 39, 45].

The frequency, duration and intensity of exercise varied across studies. All studies reported the frequency, which varied from 1 to 5 sessions per week. The exercise session duration was reported for 6 intervention groups and each exercise session lasted between 30 to 75 min [41, 42, 44, 45]. The duration of each session was constant in 4 intervention groups, whereas in 2 interventions [41, 44] it was estimated from the duration of different types of exercises that comprised the intervention. The exercise intensity was reported for 7 intervention groups [41–45]. It varied from 2.8 MET (low intensity) to 9 MET (high intensity) for each session. For all studies, PA intervention was performed according to standardized protocol followed by each participant.

#### Control Group (Comparator)

All studies compared the intervention to usual care as the control group. The number of participants in control groups varied from 19 to 89. The usual care was not described in the majority of included studies. Only the studies of Rogers et al. [45] described the usual care group. In this study, the usual care group was provided written materials related to physical activity obtained from the American Cancer Society. These materials were considered as usual care because of their availability to the general public. No specific instructions were given to the usual care group concerning PA behavior change [45].

#### Outcomes

All studies were interested in RTW as primary (n = 2) or secondary (n = 6) outcomes at different follow-up durations (endpoint). RTW was measured as event rates (binary outcome) such as rate of RTW rates in 7 studies [38–44]. One study reported Log odds as the outcome measure [45]. Outcomes were assessed at various endpoints that varied from 6 to 18 months after baseline (post-intervention). All the included studies did not report details on the meaning of RTW. Only three studies provided information about RTW as a return to full-time or part-time employment [42–44].

### Risk of Bias in Included Studies

**Fig. 2:**
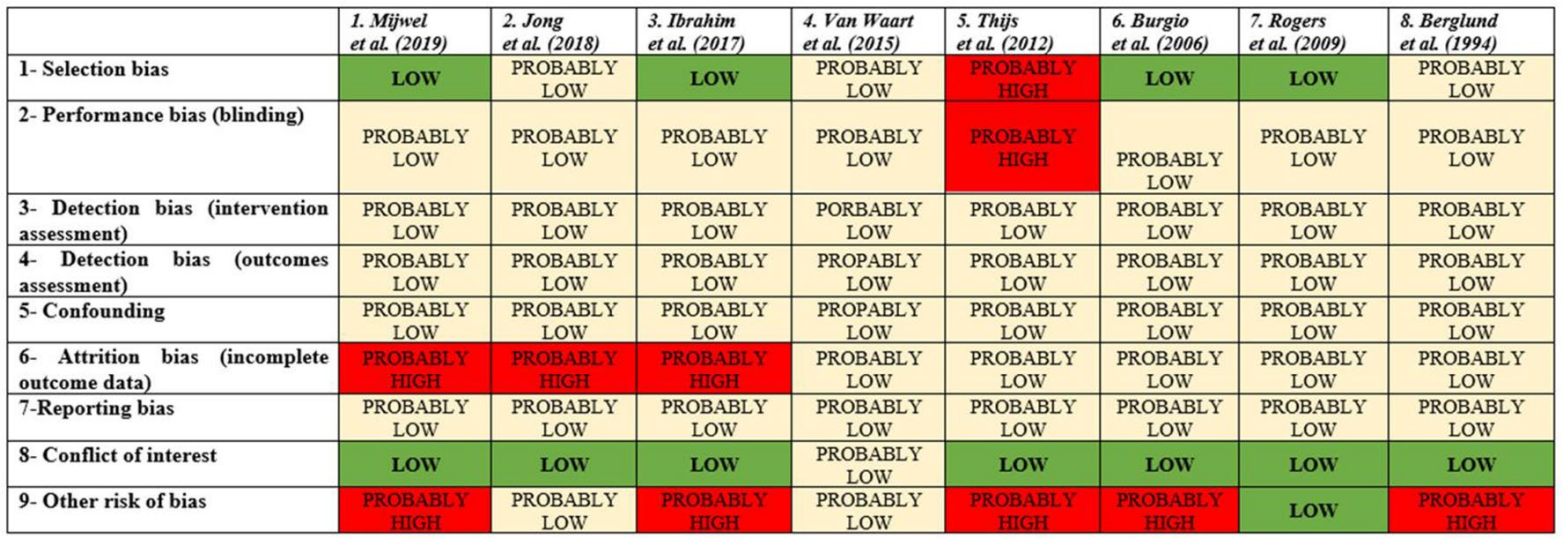
Summary of risk of bias assessment

The risk of bias assessment for each included study and by domain is summarized in Fig. 2. All studies were deemed *probably low risk* for detection biases (intervention and outcome assessment), reporting bias and confounding. This is due to the fact that PA interventions were performed by each participant following a standardized protocol and the outcomes were assessed using a standard metrics tool for all patients. Furthermore, for the risk of confounding, we considered that the randomization minimizes confounders, and this is not expected to introduce substantial bias. For the remaining domains of bias (selection bias, performance bias, attrition bias, conflict of interest, and other risk of bias), at least one of the studies was judged as being at *high risk of bias*.

For further details, the justification for each rating for each domain, by study is presented in Supplementary Table C.

### Synthesis of Results

Meta-analysis was limited to the studies that exclusively compared PA intervention to usual care. A narrative synthesis was also performed according to the results of individual studies (see Table 1).

#### Effects of PA Interventions on RTW at 12 Months: Meta-analysis

**Fig. 3:**
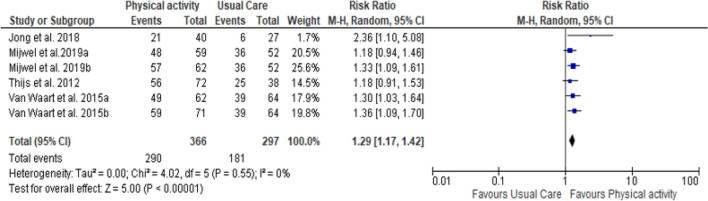
Forest plot for comparison of the effect of physical activity versus usual care

Figure 3 presents the results of meta-analysis. Due to data availability, a meta-analysis was possible only for the 4 studies that reported the rate of RTW for both the PA intervention group and control group [41–44]. Of these, 2 studies were 3-arm intervention trials [42, 44] leading to the inclusion of 6 studies in meta-analysis. Pooled estimates using random effects model showed that there is no heterogeneity among studies (P = 0.55, I^2^ = 0%). The results of meta-analysis showed a significant effect of PA interventions on RTW compared to usual care with a pooled overall RR of 1.29 (95% CI 1.17, 1.42). These results mean that PA intervention is more effective than usual care in improving RTW in cancer survivors.

#### Sensitivity Analyses

**Fig. 4:**
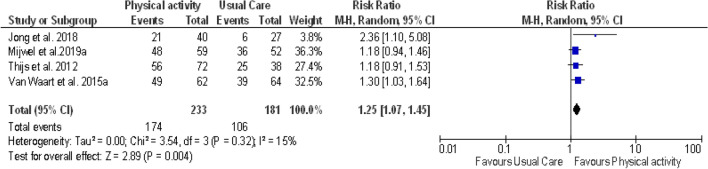
Forest plot for comparison of the effect of physical activity versus usual care (excluding AT-HIIT and OnTrack interventions groups)

**Fig. 5:**
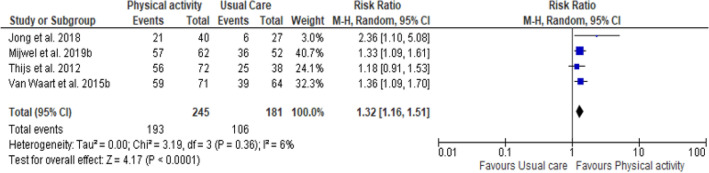
Forest plot of comparison of the effect of physical activity physical activity versus usual care (excluding RT-HIIT and OncoMove intervention groups)

Two sensitivity analyzes were performed excluding one-arm of intervention for the 3-arm RCTs. First, the intervention groups, AT-HIIT and OnTrack were excluded in the meta-analysis (see Fig. 4), and then RT-HIIT and OncoMove groups were excluded (see Fig. 5). The results always showed the lack heterogeneity among the studies with I^2^ = 15% and I^2^ = 6% respectively. They also showed a statistically significant effect on RTW in favor of PA interventions compared to usual care. The pooled RR were respectively 1.25 (95% CI 1.07, 1.45) and 1.32 (95% CI 1.16, 1.51).

#### Effects of Interventions on RTW: Narrative Synthesis

The main results from individual studies were reported in Table 1. Of the 8 included studies, 2 studies reported a statistically significant effect of PA interventions for increasing the rate of RTW [42, 44]. Findings from Mijwel et al. [42] indicated that participating in supervised aerobic exercise (in both groups: AT-HIIT and RT-HIIT) significantly increased rates of RTW (91% and 82% respectively) than usual care (69%). Similarly, results from the Van Waart et al. [44] study showed a significant increased rate of RTW for both intervention groups (OnTrack and OncoMove programs) with RTW rates of 83% and 79% compared to 61% for usual care, respectively. Authors concluded that a supervised combined aerobic and resistance exercise (OnTrack program) was the most effective in improving RTW. Likewise, 2 studies reported positive effects in favor of PA with an increased rate of RTW compared to usual care [41, 43]. The Jong et al. study [41] reported that 53% of patients were RTW at 6 months in the intervention group compared to 23% in the control group. In addition, Thijs et al. [43] found that 78% of patients in the intervention group were returned to work at 12 months compared to 68% of usual care. The Ibrahim et al. [40] study did not report the RTW rate in the control group. However, the authors concluded that the majority of participants (86%) in the intervention group returned to work.

The remaining 3 studies were multidisciplinary intervention studies that combined PA with other interventions [38, 39, 45]. Findings from these studies indicated positive effects of interventions on RTW compared to usual care. In the study by Berglund et al. [38] the rate of RTW was higher for participants in the intervention group (74.6%) compared to usual care (60.9%). Rogers et al. [45] observed an effect size of 1.49 for sick days in favor of the intervention group.

Overall, findings from included studies indicated that PA interventions improve RTW for cancer survivors compared to usual care.

#### Effects of Exercise Dose

The exercise dose was estimated from 4 studies that provided complete data for 6 interventions groups [41, 42, 44, 45] (see Table 1). The weekly exercise dose ranged from 3.55 to 15 MET.h/week. A statistically significant effect was observed on RTW for the exercise dose in 2 studies [42, 44]. The study of Mijwel et al. [42] used 2-arm intervention groups (resistance exercise and aerobic exercise) compared to usual care. The exercise dose was estimated at 7.6 METs.h/week for resistance exercise (RT-HIIT group) and 12 METs.h/week for aerobic exercise (AT-HIIT group), corresponding respectively to 60 min per session of moderate and high intensity PA twice a week. The study by Van Waart et al. [44] also included 2-arm intervention groups consisting of 30 min per session of moderate-intensity aerobic exercise 5 times per week (Onco-Move group), and 50 min per session of high-intensity combined aerobic and resistance exercise, twice a week (OnTrack group). This equated to a weekly exercise dose of 10 METs.h/wk and 15 METs.h/wk, respectively. The authors concluded that moderate to high intensity combined resistance and aerobic exercise (i.e., 15 METs.h/week) is most effective in facilitating RTW for cancer survivors.

**Fig. 6:**
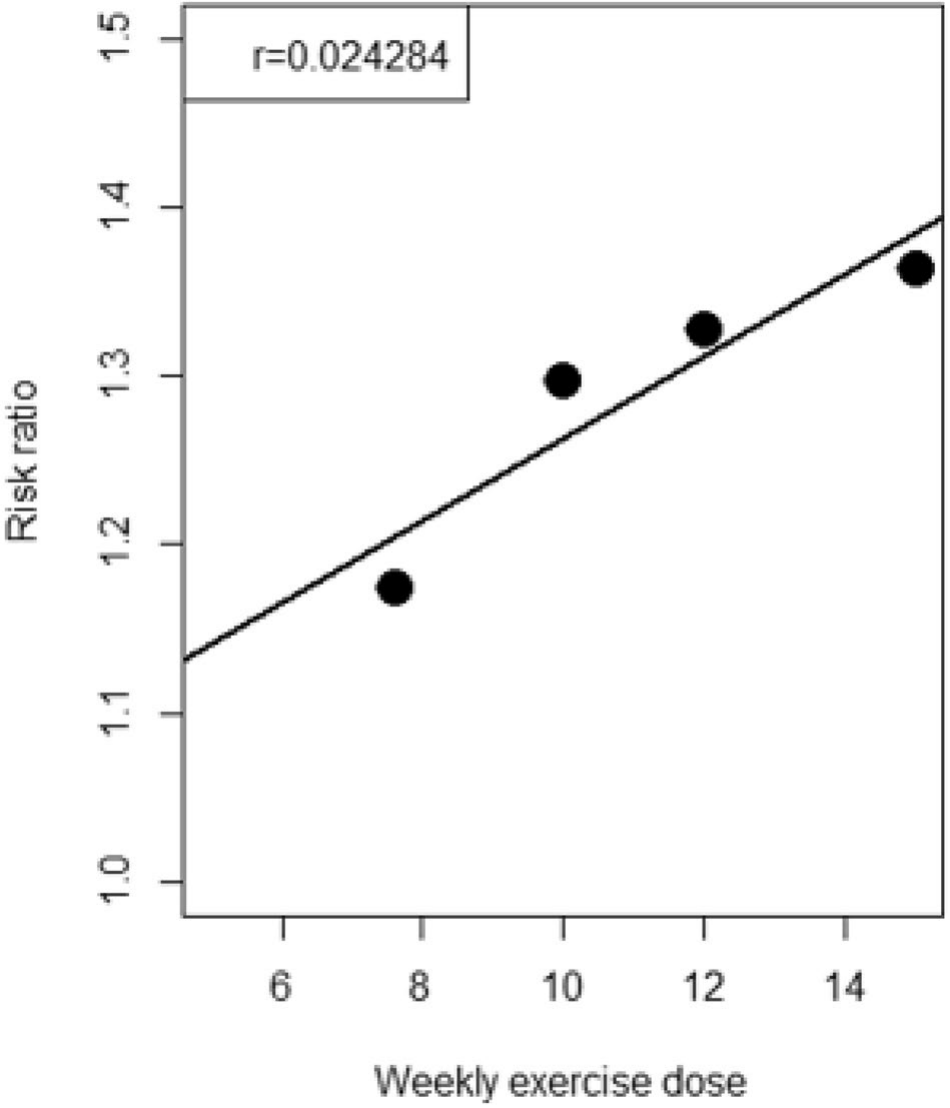
Meta-regression between risk ratio and weekly exercise dose

The meta-regression exploring the relationship between weekly dose of exercise and RR revealed a positive linear relation for RTW (regression coefficient = 0.024; P = 0.0703) (see Fig. 6). According to these results, we can suggest that effect of PA on RTW would be observed with a weekly dose of aerobic and resistance exercise between 7.6 METs.h/week and 15 METs.h/week (i.e., at least 50 to 60 min per session of moderate to high intensity PA twice a week).

### Quality of Evidence Assessment

The latest Navigation Guide instructions used by Descatha et al. [30] was adopted for grading the quality of evidence. The risk of publication bias was assessed qualitatively because the number of included studies is lower than ten.

We did not have any serious concerns regarding inconsistency, because of the lack of heterogeneity (I^2^ = 0%, P < 0.05) and the estimated RRs varied little across the studies. There were also no serious concerns regarding risk of publication bias because none of the included studies were sponsored by the industry, and we used comprehensive searches of the literature including grey literature. For indirectness, we did not have serious concerns, regarding the combination of the outcome definition, including “mixed” (rate of RTW and time to RTW), and because population, intervention and outcome did not differ from those of interest. We also had no concerns regarding imprecision given the narrow CIs in the pooled effect size estimates. Therefore, the quality of evidence was not downgraded for inconsistency, imprecision, indirectness, and risk of publication.

bias. There were very serious concerns regarding risk of bias in the body of evidence because the risk of bias was judged to be probably high, and we therefore downgraded by two levels (− 2). We did not upgrade for a large effect estimate, or for evidence for a dose–response and residual confounding. In conclusion, we started at “high” for randomized studies and downgraded by two levels (− 2) for risk of bias to a final rating of “moderate”.

## Discussion

### Main Findings

The aim of this systematic review was to assess the effects of PA intervention on RTW in cancer survivors and determine the dose of PA needed to improve this outcome. Only 8 relevant studies were included in this review, because of the paucity of PA interventions to support RTW in cancer survivors. The sample size of included studies varied from 41 to 240 for a total of 1087 participants aged between 18 and 75 years. The small sample size of the included studies may be due to the difficulties and barriers of cancer survivors to be enrolled in clinical trials [46, 47]. We included studies with participants aged up to 75 years. This is explained by the fact that the retirement age in some countries is up to 67 years [48]. Moreover, in our eligibility criteria we have taken into account the self-employed who did not have a limit for working age.

Through meta-analysis, we found a significant effect in favor of PA intervention on RTW compared to usual care. The results from narrative synthesis also revealed positive effects in favor of PA with an increased rate of RTW compared to usual care. These results could be interpreted by the moderate mediation effects of PA through the conceptual models of RTW after cancer diagnosis [10, 49–51], and by the biological effects of PA [52]. Physical activity might deal with RTW through its mediation effects on immune processes, possibly related to chronic inflammation, and its impact on psychosocial outcomes (quality of life and fatigue). Exercise intervention studies have reported results on the reduction of inflammatory biomarkers associated with cancer including C-reactive protein (CRP), interleukin-6 (IL-6) and tumor necrosis factor-α (TNF-α). Physical activity, especially resistance training, decreased leptin levels, TNF-α and insulin secretion, and increased adiponectin secretion over a seventy-two hour period, which helps to reduce chronic inflammation induced by intra-abdominal fat [53, 54]. According to conceptual models, several determinants including sociodemographic factors, disease-related factors, treatment-related factors, work-related factors, and psychosocial factors (e.g., quality of life, fatigue, and others) interact to impact the RTW of cancer survivors. There is consistent evidence that PA improves quality of life and fatigue in cancer survivors, regardless of the stage of diagnosis and treatment [55]. In addition, PA also reduces the side effects of treatment, especially deconditioning in cancer survivors by improving physical fitness [56]. Therefore, by improving these factors, PA also impacts RTW through its moderating effects. These explanations are consistent with the literature where studies showed that patients with a good quality of life returned to work earlier [5]. According to the type of cancer, most studies included breast cancer survivors, but other types of cancer comprising prostate cancer, colorectal, upper gastrointestinal, Hodgkin lymphoma and ovarian cancers were also included. The number of breast cancer studies could be explained by the fact that breast cancer is the most commonly diagnosed and prevalent cancer [1]. The included studies did not address some types of cancers (e.g., head and neck cancers, thoracic cancers, brain cancer, testis cancer, etc.). This could be explained by the fact that these cancers are rare or less frequent [1]. As stated by De Boer al.[17], is likely that the mechanisms of PA interventions are similar regardless of type of tumor, and thus cancer survivors with other types of cancer will experience the same benefits from the intervention aimed to improve RTW.

Our results revealed that the effective dose of PA on RTW in cancer survivors would be comprised between 7.6 METs.h/week and 15 METs.h/week, with an intervention duration of 16 to 20 weeks. These exercise doses respectively equate to 60 min per session of moderate-intensity aerobic exercise twice a week; and a combination of high intensity aerobic and resistance exercises, twice a week, lasting 50 min per session. The most effective exercise dose for improving RTW was 15 METs.h/week, meaning that patients who participated in supervised aerobic and resistance exercise in a hospital setting were more likely to RTW. This can be explained by the motivation of the participants when the intervention was supervised and are consistent with the findings of a recent review which showed that combined aerobic and resistance training could improve common cancer-related health outcomes [57]. Similar results were found in the study by Zopf et al. [58] which reported that both aerobic and resistance training have a positive influence on a patient's physical, psychological and social level and should therefore be included in every exercise program.

The findings of this systematic review showed that PA interventions are still scarce and there is variability across interventions. As observed in previous reviews [14–16], the interventions varied widely in content and delivery. Some interventions were performed in a hospital setting, others at home or both in hospital and at home. A marked variability was also observed in the time at which interventions were deployed and their duration. The majority were deployed after completion of cancer therapy, while the others were deployed at different stages of treatment (e.g., before, before and during, during or after treatment). These results showed that PA interventions can be delivered to cancer survivors as supportive care throughout the course of the disease (post-diagnosis). However, to be more beneficial for patients, it is recommended to start PA intervention as early as possible after cancer diagnosis [10, 57, 59]. The same observation was made regarding the mode of delivery of the intervention. Most interventions were delivered with the supervision of physiotherapists, nurses, or other health professionals. The supervision consisted of leading the intervention and providing information or counseling to the participants. The variability in the design and implementation of PA interventions makes it difficult to recommend a specific exercise protocol for cancer survivors in the RTW intervention programs. Therefore, it is challenging to offer definitive recommendations on what constitutes an effective PA intervention to support RTW for cancer survivors [15]. Additionally, studies included in this review are lacking long-term follow-up, as they did not assess the long-term effects of PA interventions (more than 2 years). In our review, the longest follow-up time reported was 18 months after intervention. Even if we found that PA intervention has positive effects on RTW, questions about the long-term effects of PA on this outcome for cancer survivors remain unanswered. Therefore, it necessary to develop further intervention studies to explore these issues.

Another pitfall of this study is the lack of uniform definition of RTW across the studies. Return to work outcomes are multifaceted; they were measured by self-reporting in all studies and varied from a continuous outcome (time to RTW) to a binary outcome (work status, work resumption, sick leave). Only 3 studies reported information on RTW as full-time or part-time work without provided more details on the meaning of RTW (i.e., return to the same job or a lesser job, to previous or new employment). The lack of clear definition of RTW could be justified by the fact that most included studies considered RTW as a secondary outcome. These results are in accordance with the literature [13, 16, 60]. As highlighted by Young et al. [61], RTW may involve returning to the pre-injury job, pre-injury employer, new employer, and work with or without accommodations as well as full-time or part-time. Therefore, it is needed for future researchers to clearly define what RTW means after cancer and choose the most suitable outcome measures.

Finally, all included studies compared the intervention to usual care as the control group. The usual care is defined as the care the targeted patient population would be expected to receive as part of the normal practice and, within RCTs, refers to the care the participants who are not receiving the tested intervention receive (i.e., without PA intervention) [62, 63]. We noticed that usual care is not the same across the studies and was not described. Yorganci et al.[62] reported that the usual care provided to patients is rarely described in detail in RCTs of a complex intervention [62].

### Strength and Limitations of This Systematic Review

This systematic review process was conducted with a pre-registered protocol and reported in accordance with the PRISMA checklist (see Supplementary Table D) to ensure methodological quality. Furthermore, we completed a comprehensive and systematic search using four data collection techniques: search in bibliographic databases, grey literature, hand search through reference lists, and expert’ consultation. This approach reduces the publication bias. We also used standard tools (Navigation Guide) specific to occupational and environmental health for assessing risk of bias in included studies. Finally, most of the included studies were RCTs, which are studies of high internal validity and constitute the gold standard to assess the effectiveness of interventions.

Like all studies, this review presents some limitations. First, the few studies included and the methodological weakness of some trials, especially the small sample sizes and lack of intention-to-treat analyzes. In addition, most of the studies included in this systematic review designed interventions without accounting factors associated with RTW such as sociodemographic and medical factors, location, and stage of tumors [64, 65]. This could explain the lack of statistically significant results in some RCTs. The final limitations concerned the generalization of results. The results of the study could not be generalized to all cancer survivors. This could be explained by the fact that most studies involved breast cancer survivors and all cancer types were not studied in our review. Additionally, the majority of patients included in the studies were those who have a sufficient physical fitness to participate in interventions, thereby excluding patients unable to participate in PA.

### Recommendation for Future Research

This systematic review showed that PA has beneficial effects on RTW for cancer survivors. However, some research questions and limitations still exist and should be considered in future research. Thus, the following recommendations are provided to design and implement an effective PA intervention to support cancer survivors to RTW:

1. Provide a clear definition of the RTW outcome that will be evaluated using the most appropriate measures according to literature [16].
2. State the details of the intervention characteristics in terms of content, such as length, setting (hospital or home), timing (i.e., related to treatment), and mode of delivery (supervised or not).
3. Specifically, interventions should be designed and reported whilst taking the FITT characteristics of PA into account (e.g., frequency, intensity, time, and type) [66], that would allow an estimation of the exercise dose.
4. More specifically, future research should implement interventions based on PA recommendations for cancer survivors and investigate the long-term effects of PA and the dose–response relationship between PA and RTW [59].
5. To avoid the methodological limitations of studies, we recommended designing them using [16, 66]:
  randomized clinical trials as the study design;
  specific eligibility criteria: clearly stated eligibility criteria;
  randomization of allocation groups (a description of the randomization method used to allocate patients into study groups should be provided);
  provide more information (detailed description) about the content of care received by the control group;
  pre-test the intervention with few participants, then pilot-test the intervention before to test the efficacy of the program;
  blinding of outcome assessors; and
  intention-to-treat analysis.

## Conclusions

In conclusion, the current study provides the description of PA intervention and a comprehensive overview of the effects of PA on RTW in cancer survivors. When summing up, PA intervention studies aimed at supporting cancer survivors to RTW remain scarce. Of the included studies, we found variability across interventions in terms of content, mode, and timing. However, our results showed with moderate quality evidence that PA interventions are more effective than usual care in increasing the rate of RTW in cancer survivors. The PA interventions (aerobic and resistance exercise) with an exercise dose between 7.6 METs.h/week and 15 METs.h/week, consisting in 50–60 min per session of moderate to vigorous physical exercise, 2 twice a week seems relevant to improve RTW. For future research, recommendations on how to design and implement PA interventions to support cancer survivors’ RTW have been proposed.

### Deviations from Protocol

Deviations from the protocol are described below:

- Concerning search strategy, some health organization websites (International Agency for Research on Cancer, European Cancer Organization and American Cancer Society) and grey literature websites (grey literature report) were not explored as we planned in the protocol.
- In the protocol, we planned to include observational studies. In the systematic review, we included only intervention studies (RCTs and nRCTs) because of their methodological quality.
- Disagreements in the study selection, data extraction, and risk of bias assessment steps were resolved by consensus rather than by a third author as specified in the protocol.

## Supplementary Information

Below is the link to the electronic supplementary material.

Supplementary file1 (DOCX 54 kb)

## Data Availability

The datasets used and/or analyzed during the current study are available from the corresponding author upon reasonable request.
