# Supplementary material for: Effectiveness of Physical Activity Interventions on Return to Work After a Cancer Diagnosis: A Systematic Review and Meta-analysis
Source: J Occup Rehabil. 2022 Jul 2;33(1):4–19. doi: 10.1007/s10926-022-10052-9 (PMC10025244; doi:10.1007/s10926-022-10052-9)
Supplement: Supplementary file 1 — Supplementary file1 (DOCX 54 kb) [file 10926_2022_10052_MOESM1_ESM.docx]

**Effectiveness of physical activity interventions on return to work after a cancer diagnosis: a systematic review and meta-analysis.**

Têtê Norbert Wilson^1^*, Aboubakari Nambiema^1^, Bertrand Porro^1^, Alexis Descatha^1^, Agnès Aublet-Cuvelier^2^, Bradley Evanoff^3^, Yves Roquelaure^1^

**Affiliations**

^1^Univ Angers, CHU Angers, Univ Rennes, Inserm, EHESP, Irset (Institut de recherche en santé, environnement et travail)- UMR_S 1085, F-49000 Angers, France

^2^INRS (Institut National de Recherche et de Sécurité), Direction des Etudes et de Recherches, 1 rue du Morvan, CS60027, 54519 Vandœuvre-lès-Nancy, France

^3^Division of General Medical Sciences, Washington University School of Medicine, St. Louis, MO 63310, USA, Saint-Louis, USA

**^*^Corresponding author:**

Têtê Norbert Wilson (PhD Student), [tetenorbert.wilson@etud.univ-angers.fr](mailto:tetenorbert.wilson@etud.univ-angers.fr), +33 6 69 59 30 44

**ORCID ID**: <https://orcid.org/0000-0003-1696-7454>

**SUPPLEMENTARY MATERIALS**

**Table A** Search strategies and results.

| **Database** | **Search string** | **Record Count 08/12/2020** | **Upgrade**  **30/09/2021** |
| --- | --- | --- | --- |
| **PubMed** | (exercise[MH] OR running[TW] OR gymnastics[TW] OR “aerobic exercise”[TW] OR “exercise therapy”[MH] OR “exercise program*”[TW] OR “sports”[MH] OR sports[TW] OR “physical activit*”[TW] OR “physical training”[TW] OR “physical exercise”[TW] OR “physical fitness”[TW] OR “breathing exercise*”[TW] OR “stretching exercise*”[TW] OR “remedial exercise*”[TW] OR (“exercise”[TW] AND “rehabilitation”[TW])) AND (“return to work”[MH] OR “back-to-work”[TW] OR “return to work”[TW] OR "work resumption"[TW] OR "work rehabilitation"[TW] OR "work reintegration"[TW] OR "work retention"[TW] OR reemployment[TW] OR “occupational medicine”[MH] OR “occupational health”[MH] OR “occupational therapy”[TW] OR “occupational medicine”[TW] OR “occupational health”[TW] OR employee[TW] OR “sick leave”[MH] OR absenteeism[MH] OR ((resume[TW] OR re-enter[TW] OR re-entry[TW]) AND (job[TW] OR work[TW] OR labour[TW]))) AND (neoplasms[MH] OR cancer*[TW] OR tumo*[TW] OR carcinoma[TW] OR lymphoma[TW] OR leukemia[TW] OR melanoma[TW] OR sarcoma[TW] OR blastoma[TW] OR oncolog*[TW]) | 191 | **210** |
| **Embase** | ('exercise'/exp OR 'running'/exp OR gymnastics:ti,ab,kw OR 'aerobic exercise':ti,ab,kw OR 'exercise therapy':ti,ab,kw OR 'exercise program*':ti,ab,kw OR sport*:ti,ab,kw OR 'physical activit*':ti,ab,kw OR 'physical training':ti,ab,kw OR 'physical exercise':ti,ab,kw OR 'physical fitness'/exp OR 'breathing exercise*':ti,ab,kw OR 'stretching exercise*':ti,ab,kw OR 'remedial exercise*':ti,ab,kw OR (execise:ti,ab,kw AND 'rehabilitation'/exp)) AND ('return to work'/exp OR 'back-to-work':ti,ab,kw OR 'work resumption':ti,ab,kw OR 'work rehabilitation':ti,ab,kw OR 'work reintegration':ti,ab,kw OR 'work retention':ti,ab,kw OR reemployment:ti,ab,kw OR 'occupational medicine':ti,ab,kw OR 'occupational health':ti,ab,kw OR 'occupational therapy':ti,ab,kw OR employee:ti,ab,kw OR 'sick leave':ti,ab,kw OR 'absenteeism'/exp OR ((resume:ti,ab,kw OR 're-enter':ti,ab,kw OR 're-entry':ti,ab,kw) AND (job:ti,ab,kw OR work:ti,ab,kw OR labour:ti,ab,kw ))) AND ('neoplasm'/exp OR cancer*:ti,ab,kw OR tumo*:ti,ab,kw OR carcinoma:ti,ab,kw OR lymphoma:ti,ab,kw OR leukemia:ti,ab,kw OR melanoma:ti,ab,kw OR sarcoma:ti,ab,kw OR blastoma:ti,ab,kw OR oncolog*:ti,ab,kw ) | 278 | **314** |
| **Cochrane Library** | 1. ((exercise):ti,ab,kw OR (running):ti,ab,kw OR (gymnastics):ti,ab,kw OR (aerobic exercise):ti,ab,kw OR “exercise program*”:ti,ab,kw OR ("exercise therapy"):ti,ab,kw OR (sport*):ti,ab,kw OR ("physical activit*"):ti,ab,kw OR ("physical training"):ti,ab,kw OR ("physical exercise"):ti,ab,kw OR ("physical fitness"):ti,ab,kw OR ("breathing exercise*"):ti,ab,kw OR ("stretching exercise*"):ti,ab,kw OR ("remedial exercise*"):ti,ab,kw OR (exercise:ti,ab,kw AND rehabilitation:ti,ab,kw))  2. ("return to work"):ti,ab,kw OR (“back-to-work”):ti,ab,kw OR (work resumption):ti,ab,kw OR (work rehabilitation):ti,ab,kw OR (work reintegration):ti,ab,kw OR (work retention):ti,ab,kw OR (reemployment):ti,ab,kw OR ("occupational medicine"):ti,ab,kw OR ("occupational health"):ti,ab,kw OR ("occupational therapy"):ti,ab,kw OR (employee):ti,ab,kw OR ("sick leave"):ti,ab,kw OR (absenteeism):ti,ab,kw  3. (resume:ti,ab,kw OR re-enter:ti,ab,kw OR re-entry:ti,ab,kw) AND (job:ti,ab,kw OR work:ti,ab,kw OR labour:ti,ab,kw)  **4. (#2 OR #3)**  #5. (neoplasms:ti,ab,kw OR cancer*:ti,ab,kw OR tumo*:ti,ab,kw OR carcinoma:ti,ab,kw OR lymphoma:ti,ab,kw OR leukemia:ti,ab,kw OR melanoma:ti,ab,kw OR sarcoma:ti,ab,kw OR blastoma:ti,ab,kw OR oncolog*:ti,ab,kw)  **6. (#1 AND #4 AND #5)** | 174 | **188** |
| **Web of Sciences** | TS=((exercise OR running OR gymnastics OR "aerobic exercise" OR "exercise therapy" OR “exercise program*” OR sport* OR "physical activit*" OR "physical training" OR "physical exercise" OR "physical fitness" OR "breathing exercise*" OR "stretching exercise*" OR "remedial exercise*" OR ("exercise" AND "rehabilitation")) AND ("return to work" OR "back-to-work" OR "work resumption" OR "work rehabilitation" OR "work reintegration" OR "work retention" OR reemployment OR "occupational medicine" OR "occupational health" OR "occupational therapy" OR employee OR "sick leave" OR absenteeism OR ((resume OR re-enter OR re-entry) AND (job OR work OR labour))) AND (neoplasms OR cancer* OR tumo* OR carcinoma OR lymphoma OR leukemia OR melanoma OR sarcoma OR blastoma OR oncolog*)) | 344 | **375** |
| **Scopus** | TITLE-ABS-KEY((exercise OR running OR gymnastics OR "aerobic exercise" OR "exercise therapy" OR“exercise program*” OR sport* OR "physical activit*" OR "physical training" OR "physical exercise" OR "physical fitness" OR "breathing exercise*" OR "stretching exercise*" OR "remedial exercise*" OR (exercise AND rehabilitation)) AND ("return to work" OR "back-to-work" OR "work resumption" OR "work rehabilitation" OR "work reintegration" OR "work retention" OR reemployment OR "occupational medicine" OR "occupational health" OR "occupational therapy" OR employee OR "sick leave" OR absenteeism OR ((resume OR re-enter OR re-entry) AND (job OR work OR labour))) AND (neoplasms OR cancer* OR tumo* OR carcinoma OR lymphoma OR leukemia OR melanoma OR sarcoma OR blastoma OR oncolog*)) | 686 | **745** |
| **Psycinfo** | (DE(exercise OR sports) OR TX(running OR gymnastics OR “aerobic exercise” OR “exercise therapy” OR “exercise program*” OR sports OR “physical activit*” OR “physical training” OR “physical exercise” OR “physical fitness” OR “breathing exercise*” OR “stretching exercise*” OR “remedial exercise*” OR (“exercise” AND “rehabilitation”))) AND (DE (“occupational therapy” OR “occupational health”) OR TX(“return to work” OR “back-to-work” OR “work resumption” OR “work rehabilitation” OR “work reintegration” OR “work retention” OR reemployment OR “occupational medicine” OR “occupational health” OR “occupational therapy” OR employee OR “sick leave” OR absenteeism OR ((resume OR re-enter OR re-entry) AND (job OR work OR labour)))) AND (DE(neoplasms) OR TX(cancer* OR tumo* OR carcinoma OR lymphoma OR leukemia OR melanoma OR sarcoma OR blastoma OR oncolog*)) | 145 | **151** |
| **Clinical Trials Gov** | ( exercise OR running OR gymnastics OR “aerobic exercise” OR “exercise therapy” OR sport* OR “physical activit*” OR “physical training” OR “physical exercise” OR “physical fitness” OR “breathing exercise*” OR “stretching exercise*” OR “remedial exercise*” OR “exercise” AND “rehabilitation” ) AND ( “return to work” OR “back-to-work” OR “work resumption” OR “work rehabilitation” OR “work reintegration” OR “work retention” OR reemployment OR “occupational medicine” OR “occupational health” OR “occupational therapy” employee OR “sick leave” OR absenteeism OR ( resume OR re-enter OR re-entry ) AND ( job OR work OR labour ) ) AND ( neoplasms OR cancer* OR tumo* OR carcinoma OR lymphoma OR leukemia OR melanoma OR sarcoma OR blastoma OR oncolog* ) AND AREA[OverallStatus] EXPAND[Term] COVER[FullMatch] ( "Recruiting" OR "Active, not recruiting" OR "Completed" OR "Enrolling by invitation" OR "Terminated" OR "Unknown status" ) | 178 | **203** |
| **Google Scholar** | (“physical activity” OR “physical training” OR “physical fitness” OR (“exercise” AND “rehabilitation”)) AND (“return to work” OR “back-to-work” OR "work retention" OR reemployment) AND (cancer OR neoplasms) / [sort by relevance](https://scholar.google.com/scholar?hl=en&as_sdt=0,5&q=%22return+to+work%22) AND exclude patents. | 200 | **200** |
| **Opengrey** | ("return to work") | 28 | **43** |
| **Osha** | ("physical activity" OR exercise) AND (cancer OR neoplasms) AND ("return to work" OR "back-to-work" OR rehabilitation) / filter on type of publication. | 45 | **78** |
| **Inca** | ("physical activity" OR exercise) AND (cancer OR neoplasms OR tumor) AND ("return to work" OR back-to-work OR rehabilitation) | 82 | **88** |
| **American Society of Clinical Oncology (ASCO)** | return to work/ filter on Abstracts/ | 46 | **52** |

**Table B** Typology and definition of physical activities components in interventions.

| **Types of Physical Activity** | **Definition** | **Example** |
| --- | --- | --- |
| **Physical activity (PA)** | is defined as any bodily movement produced by skeletal muscles that result in energy expenditure, which can be measured in kilocalories (as energy expenditure). | Exercise, sport, PA done as part of daily living, occupation, leisure, and active transportation. |
| **Exercise** | Exercise is a form of physical activity that is planned, structured, repeated and has a final or an intermediate objective to the improve or maintain physical fitness and health. | According to PA guideline for Americans***^c^***, exercises have been classified in four main types:  aerobic exercise, resistance exercise, flexibility exercise and balance. |
| **Aerobic or endurance exercise** | also called endurance exercise, it is any physical activity that uses large muscle groups and causes your body to use more oxygen than it would while resting. It causes a person’s heart to beat faster, and they will breathe harder than normal. It is the type of movement that most benefits the heart. | *Examples* of aerobic activity are running, swimming, dancing, brisk walking, jogging, and bicycling. |
| **Resistance or** **strength exercise** | also called strength training (muscles and bone strengthening), it can firm, strengthen, and tone your muscles, as well as improve bone strength, balance, and coordination. | *Examples:* exercises, bicep curls, shoulder press, bench press, barbell squat, pushups, lunges, pelvic floor muscle training, and bent over row. |
| **Flexibility or stretching exercises** | Flexibility exercises stretch your muscles and can help your body stay flexible. These activities help to improve joint flexibility and keep muscles limber, thereby preventing injury. | *Example:* yoga and Pilates |
| **Balance activity** | These kinds of exercise can improve the ability to resist forces within or outside of the body that cause falls while a person is stationary or moving. | *Example:* Walking backward, standing on one leg. |

***^C^*** The Physical Activity Guidelines for Americans were published by the United States department of health and human services. It provides information and guidance on the types and amounts of physical activity to improve a variety of health outcomes for multiple population groups.

**Table C** Details of risk of bias assessment for each included study.

| **Study ID** | **Bias** | **Domains** | **Judgements** | **Comments** |
| --- | --- | --- | --- | --- |
| **1. Mijwel et al, 2019** | *Selection bias* | Study group | **LOW** | Participants were randomly allocated to RT-HIIT, AT-HIIT, or UC using a random assignment computer program at a 1:1:1 ratio as per original paper (*Mijwel et al, 2018*). At 12 months, 95% of the women who completed pre- and post-measurements filled out all questionnaires and 78% agreed to come back for in-clinic physiological reassessments. |
|  | *Performance bias* | Blinding | PROBABLY LOW | Impossible to blind participants due to the nature of the intervention. The assignment program was blinded to the research team, prior to the first assessment. Exercise supervisors weas not masked to group allocation. The outcome was self- assessed by participants using self-reported questionnaires. According to the study design the lack of blinding did not affect outcome measure (assessed by standard metrics tool). |
|  | *Detection bias* | Intervention assessment | PROBABLY LOW | There is insufficient information about intervention assessment. However, the study design (RCT) allows for accurate assessment. PA intervention was performed **according to standardized protocol followed by each participant.** |
|  |  | Outcome assessment | PROBABLY LOW | The outcome (return to work) was self-reported using questionnaire (regarding how much sick leave the participants were taking at 12 months) **that is suggest being robust methods.** |
|  | *Confounding bias* | Confounding | PROBABLY LOW | The study did not account the confounders for secondary outcome analysis. However, **the randomization minimizes confounders, and this is not expected to introduce substantial bias**. |
|  | *Attrition bias* | Incomplete Outcome Data | PROBABLY HIGH | The missing data (32/173 = 18%) were showed in flow chat but not described in the data analysis. Small dropouts (loss to follow-up limited) and high response rate. |
|  | *Reporting bias* | Selective outcome reporting | PROBABLY LOW | There is no evidence of selective reporting. Based on the outcomes outlined in the published manuscript’s methods, all outcomes have been reported in the pre- specified way. The study was free of selective reporting. |
|  | *Conflict of interest* | Conflict of interests | **LOW** | No conflict of interest detected. The study was supported by the Swedish foundations, academy grants and by government. |
|  | *Other risk of bias* | Other Bias | PROBABLY HIGH | Data were not analyzed by ITT. |
| **2. Jong et al (2018)** | *Selection bias* | Study group | PROBABLY LOW | Participants were randomized using separate randomization lists (using blocked randomization) and assigned to four strata. Lack of information concerning the recruitment and enrollment procedures. In addition, the recruitment remained at a lower than anticipated rate and the participation rate was approximately **35%** (83/239) of eligible participants. |
|  | *Performance bias* | Blinding | PROBABLY LOW | Impossible to blind participants due to the nature of the intervention. The study monitor was blinded for allocation sequence and assigned subjects to the groups.  The outcome was self- assessed by participants using semi-structured telephone interview. **According to the study design the lack of blinding did not affect outcome measure (assessed by standard metrics tool).** |
|  | *Detection bias* | Intervention assessment | PROBABLY LOW | There is insufficient information about intervention assessment. However, the study design (RCT) allows for accurate assessment. PA intervention was performed according **to standardized protocol followed by each participant**. |
|  |  | Outcome assessment | PROBABLY LOW | The outcome (return to work) was assessed by self-reported using semi-structured telephone interview carried out by the research physician at 3 and at 6 months follow-up. **That is suggest being robust methods.** |
|  | *Confounding bias* | Confounding | PROBABLY LOW | The study did not account the confounders for outcomes analysis. However, the randomization and stratification minimize confounders, **and this is not expected to introduce substantial bias.** |
|  | *Attrition bias* | Incomplete Outcome Data | PROBABLY HIGH | Not all participants who were randomized were accounted for at 3 months. General missing data were reported in the flowchart, but no precision concerning those with the outcome and not imputed in analysis. |
|  | *Reporting bias* | Selective outcome reporting | PROBABLY LOW | No protocol published. Based on the outcomes outlined in the published manuscript’s methods, all outcomes have been reported in the pre- specified way. There is no evidence of selective reporting. |
|  | *Conflict of interest* | Conflict of interests | **LOW** | No competing financial interests exist. The study was funded by a public founding (Pink Ribbon (Grant: 2011, W016.C97). |
|  | *Other risk of bias* | Other Bias | PROBABLY LOW | More participants were randomized to the intervention group than to control group. This resulted in an imbalance of participants between the two groups. The large difference in group size probably explained the finding that the two comparison groups differed at baseline for several primary and secondary outcome parameters. |
| **3. Ibrahim et al (2017)** | *Selection bias* | Study group | **LOW** | Participants (a total of 59 young women) were equally randomized into the control (n= 30) and intervention (n= 29) arm. A block randomization process was used to generate a table through hhttp://www.randomization.com. There is sufficient information about inclusion/exclusion criteria. No statistically significant differences were observed among the intervention and control arm’s baseline characteristics. |
|  | *Performance bias* | Blinding | PROBABLY LOW | Impossible to blind participants due to the nature of the intervention. However, access to the randomization table was limited to a single individual, and the treatment assignment was hidden until a participant’s name was entered. Participants were the outcome assessors (as using self-reported post hoc questionnaire). **According to the study design that the lack of blinding did not affect outcome measure (assessed by standard metrics tool).** |
|  | *Detection bias* | Intervention assessment | PROBABLY LOW | There is insufficient information about intervention assessment. However, the study design (RCT) allows for accurate assessment. PA intervention was performed according **to standardized protocol followed by each participant**. |
|  |  | Outcome assessment | PROBABLY LOW | The outcome (return-to-work) was assessed using a post hoc questionnaire to compare participants’ pre-diagnostic work hours. |
|  | *Confounding bias* | Confounding | PROBABLY LOW | The study did not account the confounders for outcomes analysis. However, the randomization minimizes confounders, and this is not expected to introduce substantial bias. |
|  | *Attrition bias* | Incomplete Outcome Data | PROBABLY HIGH | Two dropped out and 3 died during the study. At 18 months follow-up, 8 patients **(13.56%)** have missing data because of several document events. Missing data were not included in the analysis. |
|  | *Reporting bias* | Selective outcome reporting | PROBABLY LOW | No protocol published. Based on the outcomes outlined in the published manuscript’s methods, all outcomes have been reported in the pre- specified way. There is no evidence of selective reporting. |
|  | *Conflict of interest* | Conflict of interests | **LOW** | Funding source is from non-profit organization and government agencies. Authors declare no conflict of interest. |
|  | *Other risk of bias* | Other Bias | PROBABLY HIGH | The low adherence to the use of self-reported logs. Moreover, intention-to-treat analysis was not performed. |
| **4. Van Waart et al (2015)** | *Selection bias* | Study group | PROBABLY LOW | Patients were randomly assigned to Onco-Move, OnTrack, or UC groups using the minimization method for allocation to clinical trials. **There is insufficient information concerning the recruitment and enrollment procedures (inclusion/exclusion criteria).** No significant difference in background characteristics between participants and non-participants. Participation rate **44%** (230 of 524 eligible patients). |
|  | *Performance bias* | Blinding | PROBABLY LOW | Impossible to blind participants due to the nature of the intervention. Lack of information about blinding of study investigators. Participants were the outcome assessors (as using self-reported RTW questionnaire). **According to the study design, absence of blinding judged as not significantly impacting outcome measure (assessed by standard metrics tool).** |
|  | *Detection bias* | Intervention assessment | PORBABLY LOW | There is insufficient information about intervention assessment. However, the study design (RCT) allows for accurate assessment. PA intervention was performed according **to standardized protocol followed by each participant**. |
|  |  | Outcome assessment | PROPABLY LOW | The outcome (return to work) was self-reported. Patients underwent performance-based tests and completed questionnaires at completion of chemotherapy (T1), and 6 months after completion of chemotherapy (T2). |
|  | *Confounding bias* | Confounding | PROPABLY LOW | The inclusion (randomization) minimizes the confounders and did not affect the result. |
|  | *Attrition bias* | Incomplete Outcome Data | PROBABLY LOW | All analyses were conducted on an intention-to-treat basis. However, outcome data were available for 204 participants (89%) directly after chemotherapy, and for 196 (85%) at the 6-month follow-up. Missing data not imputed. |
|  | *Reporting bias* | Selective outcome reporting | PROBABLY LOW | No protocol published. Based on the outcomes outlined in the published manuscript’s methods, all outcomes have been reported in the pre- specified way. There is no evidence of selective reporting. |
|  | *Conflict of interest* | Conflict of interests | PROBABLY HIGH | The study was supported by public agencies. However, two authors declared research funding from pharmaceutical Labs (Novartis Roche and AstraZeneca). |
|  | *Other risk of bias* | Other Bias | PROBABLY LOW | All analyses were conducted on an intention-to-treat basis. Half of the eligible patients declined to participate in the trial. This is a common finding in exercise oncology trials and raises issues regarding the generalizability of results to the larger target population. |
| **5. Thijs et al (2012)** | *Selection bias* | Study group | PROBABLY HIGH | Patients were not randomized. There is sufficient information about the recruitment and enrollment procedures (inclusion/exclusion criteria). The response rate was 95 % in the intervention group and 74% in the control group. There is no significant differences in patient characteristics at baseline between the two groups. |
|  | *Performance bias* | Blinding | PROBABLY HIGH | Impossible to blind participants due to the nature of the intervention.  No blinding, the outcome was reported by the patients (self-reported) with validated telephonic job resumption questionnaire. |
|  | *Detection bias* | Intervention assessment | PROBABLY LOW | There is insufficient information about intervention assessment. However, the study design allows for accurate assessment. PA intervention was performed by each participant follow **standardized methods and supervised**. |
|  |  | Outcome assessment | PROBABLY LOW | The outcome (return-to-work) was assessed using a telephonic job resumption questionnaire validated in research on chronic diseases and work **that is suggest being robust methods.** |
|  | *Confounding bias* | Confounding | PROBABLY LOW | The design of the study allows to minimize the confounders (**age matched controlled**). In addition, analyses were adjusted to most important confounders (age, sex, education level), but other confounders may also be relevant. |
|  | *Attrition bias* | Incomplete outcome data | PROBABLY LOW | No missing data for outcome reported and no loss of follow-up. However, **11 patients in control group were excluded from the main analysis.** |
|  | *Reporting bias* | Selective outcome reporting | PROBABLY LOW | No protocol published. Based on the outcomes outlined in the published manuscript’s methods, all outcomes have been reported in the pre- specified way. There is no evidence of selective reporting. |
|  | *Conflict of interest* | Conflict of interests | **LOW** | The study was financed by non-profit organizations. No commercial party having a direct financial interest in the study results. |
|  | *Other risk of bias* | Other Bias | PROBABLY HIGH | Difficult to generalize results to patients with other cancer because of high number breast cancer patients. Patients were not able to exactly recall the details and times of return-to-work can introduce memory bias. |
| **6. Burgio et al (2006)** | *Selection bias* | Study group | **LOW** | Participants were randomized using computer generated random numbers and a block size of 4. Participant’s selection, inclusion and exclusion criteria were described. No significant differences were found between the groups on key variables. |
|  | *Performance bias* | Blinding | PROBABLY LOW | Impossible to blind participants due to the nature of the intervention. The randomization schedule was implemented by the research nurse, so that interventionists would be blinded to the next group assignment. Participants were the outcome assessors (as using self-reported RTW questionnaire). **According to the study design, absence of blinding judged as not significantly impacting outcome measure (assessed by standard metrics tool).** |
|  | *Detection bias* | Intervention assessment | PROBABLY LOW | The study design (RCT) allows for accurate intervention assessment. PA intervention was performed by each participant following standardized methods described in the study. |
|  |  | Outcome assessment | PROBABLY LOW | The outcome (return-to-work) was self-assessed 6 weeks, 3 months and 6 months following surgery reported using a standard questionnaire that is suggest being robust methods. |
|  | *Confounding bias* | Confounding | PROBABLY LOW | Analysis did not take into account confounders. However, prior to randomization, patients were stratified by age, tumor differentiation. In addition, the study design RCT allows to minimize the confounding bias. |
|  | *Attrition bias* | Incomplete Outcome Data | PROBABLY LOW | No information was provided for patients with missing data. **The drop-out were not important (lower rate) in groups and reasons for drop-out are given.** |
|  | *Reporting bias* | Selective outcome reporting | PROBABLY LOW | No protocol published. Based on the outcomes outlined in the published manuscript’s methods, all outcomes have been reported in the pre- specified way. |
|  | *Conflict of interest* | Conflicts of interest | **LOW** | The study was supported by grant from public agencies. |
|  | *Other risk of bias* | Other Bias | PROBABLY HIGH | **An intent-to-treat analysis did not perform**. For the work-related data for people working at baseline no attrition/exclusion statistics were given. |
| **7. Rogers et al (2009)** | *Selection bias* | Study group | **LOW** | Participants were randomized after completion of all baseline assessments. Randomization was computer generated and kept in sealed envelopes until randomization to prevent bias in group allocation by study personnel. Recruitment process was described, eligibility criteria (inclusion/exclusion) were reported. No significant differences in patient characteristics at baseline between the two groups. |
|  | *Performance bias* | Blinding | PROBABLY LOW | Impossible to blind participants due to the nature of the intervention. The randomization was implemented to prevent bias in group allocation by study personnel. Participants were the outcome assessors (as using self-reported RTW questionnaire). **According to the study design, absence of blinding judged as not significantly impacting outcome measure (assessed by standard metrics tool).** |
|  | *Detection bias* | Intervention assessment | PROBABLY LOW | There is insufficient information about intervention assessment. However, the study design allows for accurate assessment, PA intervention was performed by each participant follow standardized methods **according to specific protocol**. |
|  |  | Outcome assessment | PROBABLY LOW | The outcome was self-reported (using standard self-reported questionnaires) that is suggest being robust methods. Measurement was obtained after 3 months (immediately post-intervention). |
|  | *Confounding bias* | Confounding | PROBABLY LOW | Analysis did not take into account confounders. In addition, the study design RCT allows to minimize the confounding bias. |
|  | *Attrition bias* | Incomplete Outcome Data | PROBABLY LOW | The authors provide an assessment of missing data in the statistical analysis section (for individual items on scales and imputation) and for those lost to follow-up. Attrition after randomized was 7% (3/41). Reasons for drop-out are reported. However, the numbers on each outcome are not provided in the tables so assumed all others were complete or imputed. |
|  | *Reporting bias* | Selective outcome reporting | PROBABLY LOW | No selective report suspected. All outcomes from methods were reported. |
|  | *Conflict of interest* | Conflict of interests | **LOW** | The project was supported by public academic grants and foundation. The authors did not have any relationships to disclose that would cause a conflict of interest. |
|  | *Other risk of bias* | Other Bias | **LOW** | No other source of bias was identified. **ITT analysis was performed**. |
| **8. Berglund et al (1994)** | *Selection bias* | Study group | PROBABLY LOW | Patients who accepted were randomized to the intervention group or to the control group. Efron’s method for randomization of small samples was used. Inclusion criteria were described. Participation’s response rate and reasons for non-responses were provided. 73 other patients declined participation but agreed to complete the questionnaire (**73/272 = 26.8 %**). There were no differences between the intervention and the control groups at any time assessed. |
|  | *Performance bias* | Blinding | PROBABLY LOW | Impossible to blind participants due to the nature of the intervention. Participants were the outcome assessors. There is not information about investigator blinding.  **According to the study design, absence of blinding judged as not significantly impacting outcome measure (assessed by standard metrics tool).** |
|  | *Detection bias* | Intervention assessment | PROBABLY LOW | There is insufficient information about intervention assessment. However, the study design allows for accurate assessment, PA intervention was performed by each participant follow standardized methods **according to specific protocol**. |
|  |  | Outcome assessment | PROBABLY LOW | The Outcome (sick leave) was self-assessed (using standard self-reported questionnaires) that is suggest being robust methods. Measurement was obtained at 3, 6 and 12 months (post-intervention). |
|  | *Confounding bias* | Confounding | PROBABLY LOW | Analysis did not allow to consider confounders. However, the study design RCT allows to minimize the confounding bias. |
|  | *Attrition bias* | Incomplete Outcome Data | PROBABLY LOW | No missing data for outcome reported and loss to follow-up not important/ no different. |
|  | *Reporting bias* | Selective outcome reporting | PROBABLY LOW | No selective report suspected. All outcomes from study methods are reported. |
|  | *Conflict of interest* | Conflict of interests | **LOW** | The study is supported by a grant from the Swedish foundation. |
|  | *Other risk of bias* | Other Bias | PROBABLY HIGH | ITT analysis was not performed. ANOVA scores are based on those patients who provided data for all points of assessment. |

**Table D** PRISMA 2009 Checklist.

| **Section/topic** | **Items #** | **Checklist item** | **Reported on page #** |
| --- | --- | --- | --- |
| **TITLE** | | |  |
| Title | 1 | Identify the report as a systematic review, meta-analysis, or both. | 1 |
| **ABSTRACT** | | |  |
| Structured summary | 2 | Provide a structured summary including, as applicable: background; objectives; data sources; study eligibility criteria, participants, and interventions; study appraisal and synthesis methods; results; limitations; conclusions and implications of key findings; systematic review registration number. | 3 |
| **INTRODUCTION** | | |  |
| Rationale | 3 | Describe the rationale for the review in the context of what is already known. | 4 |
| Objectives | 4 | Provide an explicit statement of questions being addressed with reference to participants, interventions, comparisons, outcomes, and study design (PICOS). | 4 |
| **METHODS** | | |  |
| Protocol and registration | 5 | Indicate if a review protocol exists, if and where it can be accessed (e.g., Web address), and, if available, provide registration information including registration number. | 5 |
| Eligibility criteria | 6 | Specify study characteristics (e.g., PICOS, length of follow-up) and report characteristics (e.g., years considered, language, publication status) used as criteria for eligibility, giving rationale. | 5 |
| Information sources | 7 | Describe all information sources (e.g., databases with dates of coverage, contact with study authors to identify additional studies) in the search and date last searched. | 5-6 |
| Search | 8 | Present full electronic search strategy for at least one database, including any limits used, such that it could be repeated. | 6 and supplementary Table A |
| Study selection | 9 | State the process for selecting studies (i.e., screening, eligibility, included in systematic review, and, if applicable, included in the meta-analysis). | 6 |
| Data collection process | 10 | Describe method of data extraction from reports (e.g., piloted forms, independently, in duplicate) and any processes for obtaining and confirming data from investigators. | 6 |
| Data items | 11 | List and define all variables for which data were sought (e.g., PICOS, funding sources) and any assumptions and simplifications made. | 6 |
| Risk of bias in individual studies | 12 | Describe methods used for assessing risk of bias of individual studies (including specification of whether this was done at the study or outcome level), and how this information is to be used in any data synthesis. | 7 |
| Summary measures | 13 | State the principal summary measures (e.g., risk ratio, difference in means). | 7 |
| Synthesis of results | 14 | Describe the methods of handling data and combining results of studies, if done, including measures of consistency (e.g., I^2^) for each meta-analysis. | 8 |

| **Section/topic** | **Items #** | **Checklist item** | **Reported on page #** |
| --- | --- | --- | --- |
| Risk of bias across studies | 15 | Specify any assessment of risk of bias that may affect the cumulative evidence (e.g., publication bias, selective reporting within studies). | 8 |
| Additional analyses | 16 | Describe methods of additional analyses (e.g., sensitivity or subgroup analyses, meta-regression), if done, indicating which were pre-specified. | 8 |
| **RESULTS** | | |  |
| Study selection | 17 | Give numbers of studies screened, assessed for eligibility, and included in the review, with reasons for exclusions at each stage, ideally with a flow diagram. | 8-9 and Fig. 1 |
| Study characteristics | 18 | For each study, present characteristics for which data were extracted (e.g., study size, PICOS, follow-up period) and provide the citations. | 9-10 and  Table 1 |
| Risk of bias within studies | 19 | Present data on risk of bias of each study and, if available, any outcome level assessment (see item 12). | 10-11 |
| Results of individual studies | 20 | For all outcomes considered (benefits or harms), present, for each study: (a) simple summary data for each intervention group (b) effect estimates and confidence intervals, ideally with a forest plot. | 11 |
| Synthesis of results | 21 | Present results of each meta-analysis done, including confidence intervals and measures of consistency. | 11 |
| Risk of bias across studies | 22 | Present results of any assessment of risk of bias across studies (see Item 15). | 11 |
| Additional analysis | 23 | Give results of additional analyses, if done (e.g., sensitivity or subgroup analyses, meta-regression [see Item 16]). | 12-13 |
| **DISCUSSION** | | |  |
| Summary of evidence | 24 | Summarize the main findings including the strength of evidence for each main outcome; consider their relevance to key groups (e.g., healthcare providers, users, and policy makers). | 13-15 |
| Limitations | 25 | Discuss limitations at study and outcome level (e.g., risk of bias), and at review-level (e.g., incomplete retrieval of identified research, reporting bias). | 15-16 |
| Conclusions | 26 | Provide a general interpretation of the results in the context of other evidence, and implications for future research. | 16-17 |
| **FUNDING** | | |  |
| Funding | 27 | Describe sources of funding for the systematic review and other support (e.g., supply of data); role of funders for the systematic review. | 1 |
